# Improving the Antioxidant Activity and Consumer Acceptance of Rice Snacks Fortified With Barley

**DOI:** 10.1111/1750-3841.71302

**Published:** 2026-08-19

**Authors:** Il Sook Choi, Eun Jung Kwak

**Affiliations:** ^1^ Department of Food and Nutrition Wonkwang University Iksan Jeonbuk Republic of Korea; ^2^ Institution for Better Living Wonkwang University Iksan Republic of Korea; ^3^ Department of Food Science & Technology Yeungnam University Gyeongsan Republic of Korea

**Keywords:** antioxidant activity, barley, consumer acceptance, ferulic acid, phenolic profile, rice snack

## Abstract

Although ricebased snacks are popular, they are primarily composed of refined starch and often lack dietary fiber and bioactive compounds. Barley is rich in these compounds; however, its incorporation into rice‐based products often results in undesirable changes in physicochemical and sensory properties. Therefore, this study aimed to evaluate the effects of barley fortification on physicochemical properties and antioxidant activities, as well as consumer acceptance of rice snacks to identify an appropriate barley percentage. Rice snacks containing different barley percentages (0%, 20%, 40%, 60%, 80%, and 100%) were analyzed for physicochemical properties, phenolic composition, antioxidant activities, and consumer acceptance. Total phenolics, flavonoids, and phenolic acids, as well as antioxidant activities (DPPH, ABTS, FRAP, and reducing power) increased significantly with increasing barley percentage. Ferulic acid was identified as the predominant free phenolic compound, ranging from 41.77 to 116.99 µg/g. The increase in phenolic acids in rice snacks with higher barley percentages may be associated with release of bound phenolics during high‐temperature, short‐time processing, as well as concentration effects due to moisture loss. Consumer acceptance was highest at barley percentages between 40 and 60% (6.21–6.49), although further increases in barley percentage negatively affected textural and sensory attributes, including hardness and color. In conclusion, increasing barley percentage enhances the phenolic composition and antioxidant activities of rice snacks, with changes in physicochemical and sensory attributes. A barley percentage range of 40%–60% appears to provide an appropriate balance between functional enhancements and consumer acceptance.

**Practical Applications:**

This study provides a practical formulation for manufacturing nutrient‐enriched rice snacks by identifying that a 40–60% barley fortification range provides an optimal balance between antioxidant functionality and high consumer acceptance. These findings can be directly utilized by the snack food industry to develop health‐oriented functional grain products with improved phenolic composition and enhanced sensory attributes.

## 1 Introduction

In contemporary fast‐paced lifestyles, snacks have become an essential component of daily diets (Millar et al. 2017). The global snack market has demonstrated steady growth from 2016 through 2025 (Balan et al. 2021). In 2025, the market was valued at $280.24 billion and is projected to reach $344.76 billion by 2030, representing a 4.23% compound annual growth rate (CAGR) over the forecast period (Mordor Intelligence 2025). Despite their limited nutritional value and high‐calorie content, oil‐fried savory snacks such as potato chips and pretzels continue to achieve strong market performance (Balan et al. 2021). In 2024, salty snacks comprised 32.87% of the total global snack market (Mordor Intelligence 2025). However, increased awareness of the association between salty snack intake and health concerns, including hypertension, diabetes, and obesity, has been highlighted (Balan et al. 2021), and nutritional as well as health attributes now play an important role in consumer preference (Millar et al. 2017).

Rice (*Oryza sativa* L.) serves as a fundamental staple food for more than half of the world's population, providing a primary source of energy and essential nutrients (Muthayya et al. 2014). While commonly consumed as whole kernels, rice usage in processed products—such as rice noodles, breakfast cereals, gluten‐free pasta, rice flakes, and crackers—is also on the rise (Muthayya et al. 2014). The chemical composition of rice changes in accordance with the variety, milling rate, storage period, and cultivation region. Typically, rice comprises over 77%–89% starch, 6.3%–7.1% protein, 0.3%–0.5% fat, and trace amounts of vitamins, minerals, and bioactive components, including phenolic acids, polyphenolics, gamma‐oryzanol, tocopherol, and fiber (Juliano and Hicks 1996; Ma et al. 2022). Of particular note, phenolic compounds demonstrate antioxidant and anti‐inflammatory activities. These active compounds are primarily located in rice bran and are linked to a decreased risk of chronic diseases, such as cardiovascular disease, and to alleviation of type 2 diabetes symptoms (Ma et al. 2022).

Barley (*Hordeum vulgare* L.) is a highly significant cereal crop grown globally under diverse environmental conditions, spanning both temperate and subtropical regions (Goudar et al. 2020). In 2013, global barley production totaled 145 million tons, ranking it as the fourth most produced cereal crop. The hulled barley variety is most widely cultivated, predominantly for malting, brewing, and use as animal feed. Conversely, hull‐less barley, characterized by an easily removable hull, is preferred for human dietary purposes (Goudar et al. 2020). Barley is recognized as a health‐promoting staple food due to its abundant nutrients, which includes 50.36%–72.46% starch, 7%–25% protein, along with vitamins, minerals, and various bioactive components including β‐glucan, phenolic acids, and flavonoids (Geng et al. 2022; Raj et al. 2023). These phenolics in cereal grains can be found in three forms—free, soluble conjugated, and insoluble bound compounds, with the majority existing as the bound fraction linked to fiber in the cell wall (Raj et al. 2023). However, the digestion of this cell wall material by endogenous enzymes in the small intestine is difficult. Therefore, releasing insoluble bound phenolics before consumption is essential to maximize their health benefits. Thermal processing, such as baking, is a well‐known and effective method to release bound phenolics in barley (Sharma and Gujral 2014). Thus, based on previous studies identifying various hydroxybenzoic and cinnamic acid derivatives (Goufo and Trindade 2014; Geng et al. 2022; Raj et al. 2023) as major bioactive constituents, this study investigated the representative phenolic acids commonly reported in rice and barley to evaluate the impact of such processing on their profiles.

Studies conducted over the last few years on snacks have focused on incorporating ingredients rich in functional components, such as nutraceuticals and antioxidants (Balan et al. 2021; Punia Bangar et al. 2022; Giannoutsos et al. 2023). Ongoing studies aim to partially or fully substitute wheat with alternative grains that exhibit improved functional characteristics, including rice, barley (Sharma and Gujral 2014; Punia Bangar et al. 2022; Giannoutsos et al. 2023), and millet in various snacks. Especially, the ability to finely mill rice like wheat flour facilitates uniform blending with other ingredients, making it a suitable material for the manufacture of various snack products (Dalbhagat et al. 2019). Accordingly, with its application in gluten‐free as well as health‐oriented products, the global rice snack market is undergoing rapid evolution (Market Reports World 2025). However, compared to wheat‐based snacks, research on the physicochemical, functional, and sensory profiles of rice snacks remains insufficient, with scientific literature on their specific bioactive compounds and antioxidant activities being strictly constrained.

Unlike rice snacks such as crackers (Mir et al. 2017; Rico et al. 2019), cake (Lang et al. 2020), and extruded snack (Blandino et al. 2022), which are made by mixing rice flour and water, the sample rice snacks were produced by heating cooked rice at a high temperature for a short duration. This production process removes most of the moisture, resulting in an increased concentration of bioactive compounds, including phenolic compounds. When cooked rice is further heated until nearly all moisture evaporates, processes such as gelatinization and the Maillard reaction occur, leading to distinct changes in the sensory profile, including taste, aroma, and color (Hwang and Moon 2021). Therefore, evaluating the effects of varying barley percentages on the physicochemical properties and antioxidant activities, as well as consumer acceptance, is important for developing acceptable alternative rice snacks. The objective of this study is to establish appropriate conditions for producing barley‐fortified rice snacks, enhancing antioxidant activity while meeting consumer acceptance in response to the increasing demand for functional snack products. To achieve this, the effects of the barley percentage on physicochemical properties, phenolic composition, antioxidant activities, and consumer acceptance were investigated.

## 2 Materials and Methods

### 2.1 Chemicals and Reagents

Folon–Ciocalteu's phenol reagents, ferric chloride hexahydrate, 2,2‐diphenyl‐picrylhydrazyl (DPPH), gallic acid, 2,2′‐azinobis (3‐ethylbenzothiazoline‐6‐sulfonic acid) (ABTS), 2,4,6‐tri (2‐pyridyl)‐**S**‐triazine (TPTZ), and 6‐hydroxy‐2,5,7,8‐tetramethylchroman‐2‐carboxylic acid (Trolox) were purchased from Sigma–Aldrich (St. Louis, MO, USA). Standards for five phenolic acids of *p*‐hydroxybenzoic, syringic, caffeic, *p*‐coumaric, and ferulic acids were obtained from Sigma Chemical Co. (St. Louis, MO, USA). HPLC‐grade acetonitrile, methanol, and water were obtained from Duksan (Seoul, Korea). All chemicals and reagents were of analytical grade. Milli‐Q deionized water was used throughout the study.

### 2.2 Preparation of Rice Snacks Fortified With Barley

**FIGURE 1:**
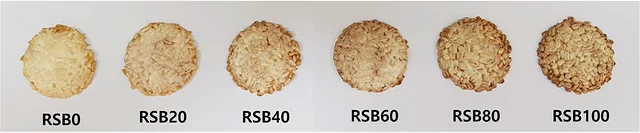
Photos of rice snacks fortified with barley. RSB0, RSB20, RSB40, RSB60, RSB80, and RSB100: Rice snacks fortified with **0%, 20%, 40%, 60%, 80%, and 100%** barley.

Both the rice and barley used in this study were sourced from Iksan, Jeollabuk‐do, Republic of Korea. Specifically, 1 kg each of rice and waxy barley (*H. vulgare* L.) were washed with water at twice their volume for 30 s, repeated five times in total. During the third wash, the grains were rubbed thoroughly to enhance cleaning. After the washing process, the grains were refrigerated for 1 h to drain residual moisture. Rice and barley were mixed at percentages of 0%, 20%, 40%, 60%, 80%, and 100%, and purified water was added at 1.2 times the combined grain weight. An electric rice cooker (HRC‐NMI0601, Cuchen Co. Ltd., Seoul, Korea) was utilized to cook the grain mixture. To produce the rice snacks, 10 g of cooked rice and barely was placed on each of the eight plates on a dual‐plate contact heating device (BE‐5200, BETHEL‐COOK Inc., Hwaseong, Korea). The device was preheated for 7 min prior to use. Upon closing the device, the rice was compressed between the upper and lower heated plates at approximately 205°C for 4 min. The resulting snacks were disk‐shaped, with a diameter of 53.39 ± 0.88 mm and a thickness of 3.06 ± 0.13 mm across all samples to ensure consistent physical properties for hardness and sensory evaluation. The resulting rice snack was ground to a particle size below 500 µm using a grinder (PULVERISETTE 11, Fritsch.de, Idar‐Oberstein, Germany) and a mortar and pestle, then stored in a freezer until analysis. The six types of rice snack samples are shown in Figure 1.

**FIGURE 2:**
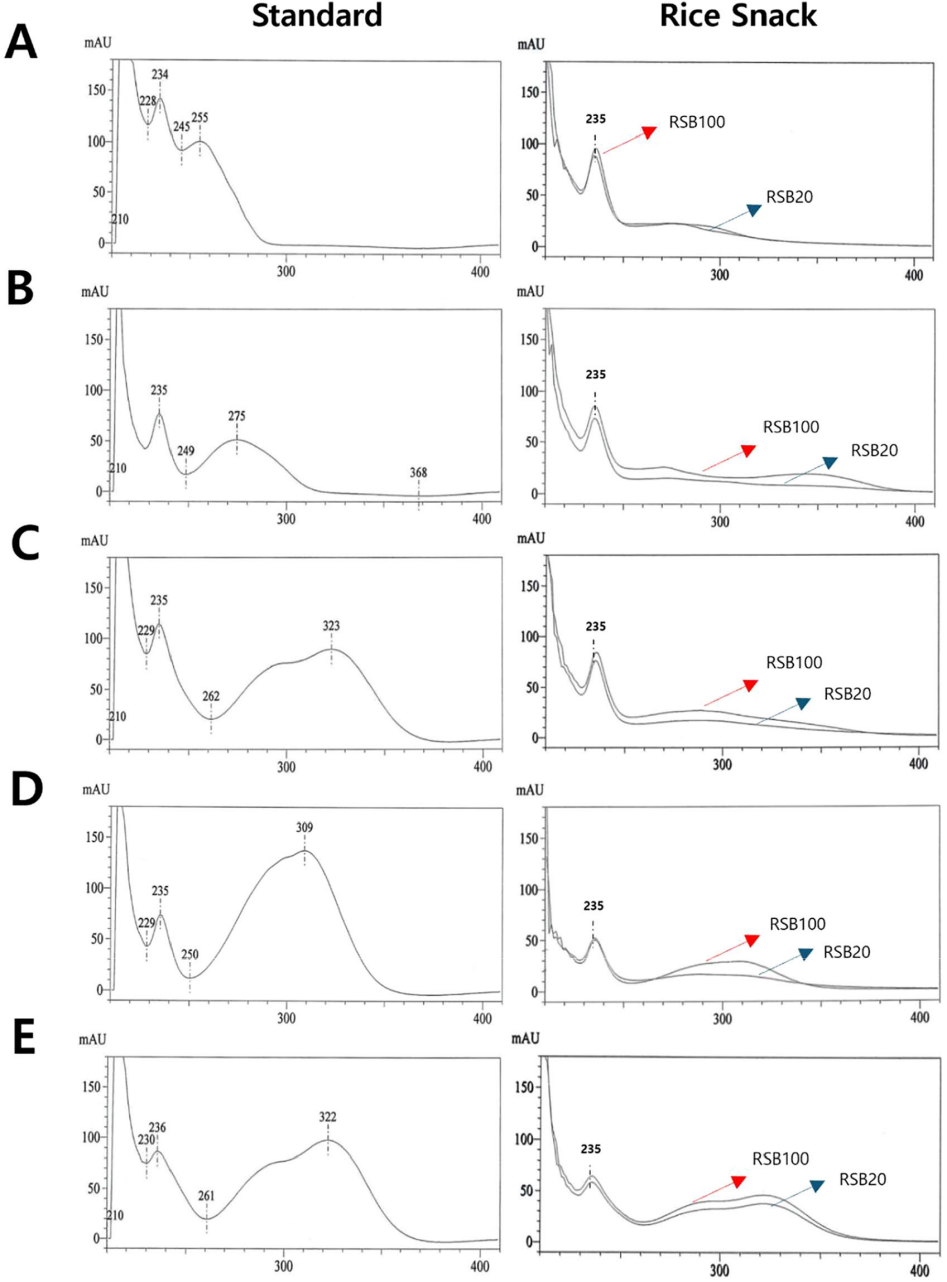
UV absorption spectra of five phenolic acid standards and rice snacks fortified with barely (RSB20 and RSB100). Phenolic acid standard concentration was set at 5 ppm. RSB20, rice snack fortified with 20% barely; RSB100, rice snack fortified with 100% barely. (A) *p*‐hydroxybenzoic acid; (B) syringic acid; (C) caffeic acid; (D) *p*‐coumaric acid; (E) ferulic acid.

### 2.3 Sample Preparation

A 10% (w/v) aqueous extract was prepared by dissolving 10 g of rice snack powder in distilled water and adjusting the final volume to 100 mL. The mixture was centrifuged at 1500 × *g* for 5 min, followed by 24,000 × *g* for 20 min. The resulting supernatant was then used to determine physicochemical properties, total phenol and flavonoid contents, and antioxidant activities.

### 2.4 Physicochemical Properties

The measurement of moisture and crude ash content was carried out according to the AOAC method (2000). Moisture content was determined by drying the samples to a constant weight in a hot air oven at 105°C using a 1 g sample. The total soluble solids content was determined using an electronic refractometer (SCM‐1000, HM Digital Inc., Seoul, Korea) with a 200 µL sample. Crude ash content was determined using the dry ashing method. The texture parameters of hardness and cohesiveness of the snack samples were measured in triplicate with Texture Analyzer (TAXT2 Express, Stable Micro Systems Ltd., Godalming, UK). Samples were positioned on the instrument platform and analyzed using a 5 mm cylindrical probe (P/25P) attached to a 20 mm probe adapter (AD/20). Parameters used in the tests were: pre‐test speed = 5.0 mm/s; trigger force 5.0 g; test speed = 5.0 mm/s; return speed = 5.0 mm/s; test distance 25 mm. Force measurements were recorded under compression mode. The pH was measured using a pH meter (S220‐K, Mettler Toledo International, Inc., Seoul, Korea). Color measurement was conducted by evenly spreading 2 g of snack powder in a Petri dish (35 × 10 mm) and measuring with a colorimeter (CR‐10 Plus, Konica Minolta Holdings, Inc., Tokyo, Japan). For calibration, the L* value of the white plate was 97.5, a* value was −0.5, and b* value was 3.0. The color differences (∆E*) between the control and the samples were calculated using the following equation: ∆E* = [(∆L*)^2^ + (∆a*)^2^ + (∆b*)^2^]^(1/2)^

### 2.5 Total Phenolic and Flavonoid Contents and Antioxidant Activities

For the measurement of total phenolic and flavonoid contents and antioxidant activities, a 10% (w/v) sample solution was centrifuged at 4000 rpm for 5 min and subsequently at 16,000 rpm for 20 min using a centrifuge. Total polyphenol content was determined following a modified protocol from Dewanto et al. (2002). To 500 µL of sample, 50 µL of Folin–Ciocalteu's reagent was added and allowed to react for 3 min, followed by the addition of 1 mL of 2% Na_2_CO_3_. The mixture was incubated in a dark room for 30 min, and the absorbance was then measured at 750 nm with a spectrophotometer (UV‐1800, Shimadzu, Tokyo, Japan). The absorbance values were quantified using a standard curve generated with gallic acid, and reported in mg gallic acid equivalent (GAE)/g.

Total flavonoid content was analyzed using a modified method described by Shen et al. (2009). To 2 mL of sample, 75 µL of 5% NaNO_2_ was added and incubated for 5 min, then 150 µL of 10% AlCl_3_·6H_2_O was introduced and allowed to react for 6 min. Subsequently, 500 µL of 1 M NaOH was added, and the mixture was kept in the dark for 11 min. Absorbance was measured at 415 nm and calculations were made with a standard curve for rutin. Results were expressed in mg rutin equivalent (RE)/g.

DPPH radical scavenging activity was assessed by a modified method of Brand‐Williams et al. (1995). The DPPH reagent (0.2 mM) was prepared and its absorbance adjusted to 1.0 with methanol. A mixture of 100 µL sample and 1 mL DPPH reagent was incubated in the dark for 30 min, after which absorbance was measured at 517 nm using a spectrophotometer. The scavenging activity was determined as a percentage using the absorbance of a blank, where 100 µL of distilled water was used instead of the sample with the DPPH reagent.

ABTS radical scavenging activity was determined using a modified protocol from Re et al. (1999). Equal volumes of 7 mM ABTS and 2.4 mM potassium persulfate were combined and incubated in the dark for 12 h to generate ABTS^+^. PBS (phosphate‐buffered saline) was added to adjust the formed solution's absorbance to 0.7. A mixture of 100 µL sample and 1 mL ABTS was incubated in the dark for 30 min, followed by absorbance measurement at 735 nm. The results were quantified according to a standard curve constructed with Trolox and values expressed as mg Trolox equivalent (TE)/g.

The FRAP working solution was prepared as described by Benzie and Strain (1996), by mixing 0.2 M sodium acetate buffer, 10 mM TPTZ, 20 mM ferric chloride hexahydrate, and distilled water at a 10:1:1:1 ratio and heating the mixture in a water bath at 37°C for 30 min. A mixture of 100 µL sample and 1 mL FRAP working solution was then incubated in the dark for 30 min, and absorbance was read at 595 nm using a spectrophotometer. The absorbance readings were converted into concentration values using a Trolox standard curve and values expressed as mg TE/g.

Reducing power was evaluated using the Oyaizu method (1986), with the pH of the 0.2 M sodium phosphate buffer adjusted to 6.6. A reaction mixture containing 100 µL sample, 300 µL of 0.2 M sodium phosphate buffer, and 300 µL of 1% potassium ferricyanide was incubated in a 50°C‐water bath for 20 min. Subsequently, 300 µL of 10% trichloroacetic acid and 100 µL of 0.1% ferric chloride were added, and the absorbance was measured at 700 nm using a spectrophotometer. The resulting absorbance values were calculated based on the standard curve equation generated for Trolox and values expressed as mg TE/g.

### 2.6 Phenolic Acid Composition

#### 2.6.1 Sample Extraction

Phenolic acids in rice snack were extracted following the method described by Ding et al. (2019). Rice snack (3.5 g) was milled using a grinder to obtain particles smaller than 30‐mesh. To 3.0 g of rice snack flour, 15 mL of 80% (v/v) methanol solution was added and mixed at room temperature on a shaker (SLRM‐3, SeouLin Bioscience, Seongnam, Korea) for 1 h at 100 rpm. The mixture was centrifuged for 5 min at 3500 rpm (FLETA5, Hanil, Daejeon, Korea). The extraction was performed three times in total. Supernatants from each extraction were pooled and evaporated to dryness using a rotary evaporator (A‐1000S, EYELA, Tokyo Rikakikai Co. Ltd, Tokyo, Japan) at 45°C, after which the residue was dissolved in 1.5 mL of methanol.

#### 2.6.2 HPLC

Phenolic acids were identified according to the procedure reported by Vasco et al. (2009), with minor modifications. Chromatographic profiling of phenolic compounds was conducted using a Shimadzu Prominence HPLC system equipped with a photodiode array detector (SPD‐M20A), oven (CTO‐20A), system controller (CBM‐20A), and automatic sampler (SIL‐20A). Separation was achieved on a Syncronis C18 column (250 mm x 4.6 mm id, particle size 5 µm) with a mobile phase comprising 1% formic acid (A solution) and acetonitrile (B solution). The gradient elution program was as follows: 0 min, 5% B; 20 min, 30% B; 31 min, 5% B. Following the run, the column was washed and equilibrated to initial conditions for 10 min. A 20 µL was injected, with a flow rate of 0.5 mL/min at 35°C. Detection of phenolic acids was performed at 280 and 320 nm, and compounds were identified by their retention time (RT), maximum absorbance wavelength, and comparison of absorption spectra at 200–400 nm with authentic standards in rice snack. Based on previous studies of rice and barley, eight phenolic acid standards were initially evaluated, namely hydroxybenzoic, dihydroxybenzoic, syringic, coumaric, caffeic, chlorogenic, sinapic, and ferulic acids (Goufo and Trindade 2014; Geng et al. 2022; Raj et al. 2023). Among these, five phenolic acids (hydroxybenzoic, syringic, coumaric, caffeic, and ferulic acids) whose RT and absorption spectra matched those of the standards were used for the final analysis. The concentration of phenolic compounds was expressed as mg of each individual compound per gram of dry weight (DW). In addition, NFB80 and NFB100 extracts used in the analysis were diluted 2‐fold with methanol to ensure accurate pipetting and consistency in measurements. Other samples were analyzed without further dilution as their fluidity was sufficient for direct handling.

### 2.7 Consumer Acceptance Test

The consumer acceptance test was conducted with official institutional clearance from the Institutional Review Board (IRB) at Wonkwang University (Approval No. WKIRB‐202408‐HR‐054). Prior to the test, every participant provided written informed consent and was screened for food allergies. A total of 108 young consumers (60 females and 48 males; aged 20 to 29 years old, *M* = 22.2, SD = 2.0) were recruited through email and flyers. All participants reported that they had previously consumed rice snacks. The consumers showed varying levels of interest in crunchy food: 17 indicated high interest, 50 indicated interest, 38 indicated indifference, and 3 indicated no interest.

Sensory evaluation took place in laboratory booths designed to minimize external disturbances, and sessions were scheduled at 10 a.m., 2 p.m., and 3 p.m., each lasting 40 min. Prior to evaluation, participants received a brief explanation regarding the procedures and assessment methods. Each participant received approximately 10 g (one piece of rice snack) served in sensory cups (7 cm × 3 cm), accompanied by water for palate cleansing during the evaluation. Each cup was marked with a three‐digit code generated from a random number table, and sample presentation order was randomized using the William Latin square design. After completing a questionnaire on general characteristics, consumers evaluated appearance acceptance, flavor acceptance, texture acceptance, after‐taste acceptance, overall acceptance, and purchase intent for six different samples (RSB0, RSB20, RSB40, RSB60, RSB80, and RSB100). Acceptance measures were rated using a 9‐point hedonic scale (0 = *extremely disliked*, 5 = *neutral*, 9 = *extremely liked*), while purchase intention was rated using either a 5‐point scale (0 = *would definitely not purchase*, 3 = *neutral*, 5 = *would definitely purchase*) or a 5‐point Likert scale.

### 2.8 Statistical Analysis

All experimental samples were tested in quadruplicate, with the results presented as mean±standard deviation using XLSTAT (Lurnivero, Co., Denver, CO, USA). Physicochemical and antioxidant properties were assessed using ANOVA (analysis of variance) at *p* = 0.05 to identify significant differences. Duncan's multiple range test was subsequently applied to compare means for significant differences. The general characteristics of consumers were analyzed using XLSTAT (Lurnivero, Denver, Co., USA), and ANOVA at *p* < 0.05 was conducted to assess differences in consumer acceptance and purchase intention across samples. Duncan's multiple range test was again employed to confirm significant differences between means. Principal component analysis (PCA) was used not only to explore associations between the samples and sensory attributes but also to examine the relationship between the samples and sensory attributes. Agglomerative Hierarchical Clustering (AHC) was conducted to segment consumers into several groups to similar samples.

## 3 Results and Discussion

### 3.1 Physicochemical Characteristics

The physicochemicl characteristics of sample rice snacks are presented in Table 1. The moisture content was highest in rice snacks without barley (RSB0) and those containing 20% (RSB20) and 40% barley (RSB40), ranging from 0.38% to 0.39%, with no significant difference between the three samples. The moisture content decreased significantly as the proportion of barley increased, with RSB100, which is composed entirely of barley, exhibiting the lowest moisture content at 0.32%. Hwang et al. (2020) found that when rice snack was prepared using varying heating durations from 2 to 14 min at 205°C, the moisture content was reduced from 3.87% at 5 min to 0.78% at 8 min, and further to 0.24% at 14 min. These findings suggest that both heating time and temperature substantially influence the moisture content of rice snacks. In the present study, sample rice snacks were heated for 4 min at 205°C, resulting in a moisture content comparable to that of rice snacks heated for 5 min, as reported by Hwang et al. (2020). The moisture content of the snacks in this study was lower than that reported in previous literature. RSB0 exhibited a significantly lower moisture content (0.40%) than rice crackers reported by Rico et al. (2019) (8.32%), which may be attributed to the limited sample mass (10 g) that facilitated efficient moisture removal during compression and heating.

**TABLE 1:** Physicochemical properties of rice snacks fortified with barley.

	RSB0	RSB20	RSB40	RSB60	RSB80	RSB100
Moisture content(%)	0.40 ± 0.02^a^	0.39 ± 0.02^a^	0.38 ± 0.01^a^	0.34 ± 0.01^b^	0.34 ± 0.01^b^	0.32 ± 0.02^b^
Total soluble solids (°Brix)	0.30 ± 0.00^f^	0.60 ± 0.00^e^	0.73 ± 0.06^d^	0.90 ± 0.00^c^	1.00 ± 0.00^b^	1.20 ± 0.00^a^
pH	6.58 ± 0.01^a^	6.21 ± 0.01^b^	5.95 ± 0.00^c^	5.66 ± 0.00^d^	5.32 ± 0.01^e^	5.12 ± 0.00^f^
Ash content (%)	0.24 ± 0.01^e^	0.24 ± 0.00^e^	0.38 ± 0.00^d^	0.42 ± 0.00^c^	0.52 ± 0.00^b^	0.61 ± 0.01^a^
Hardness (N)	5378.97 ± 107.15^a^	8517 ± 420.96^b^	6888.53 ± 102.03^c^	10116.98 ± 236.61^d^	10216.67 ± 359.35^de^	11040.91 ± 430.10^e^
L* value	70.67 ± 0.98^a^	68.38 ± 0.64^b^	68.28 ± 0.24^b^	67.92 ± 0.73^b^	66.42 ± 0.39^c^	65.60 ± 0.33^c^
a* value	3.71 ± 0.30^d^	4.06 ± 0.19^c^	4.11 ± 0.06^bc^	4.16 ± 0.01^bc^	4.38 ± 0.03^ab^	4.47 ± 0.12^a^
b* value	14.38 ± 0.25^a^	12.95 ± 0.35^b^	12.67 ± 0.39^bc^	12.28 ± 0.20^cd^	12.19 ± 0.37^cd^	11.98 ± 0.18^d^
𝚫*E*	0.77 ± 0.47^c^	2.74 ± 0.65^b^	2.97 ± 0.38^b^	3.50 ± 0.69^b^	4.83 ± 0.51^a^	5.66 ± 0.31^a^

*Note*: Results are expressed as mean ± standard deviation (*n* = 3). Different letters in the same row indicate significant differences (*p* < 0.05). L*, lightness; a*, redness; b*, yellowness; Δ*E*, color difference. RSB0, RSB20, RSB40, RSB60, RSB80, and RSB100: Rice snacks fortified with 0%, 20%, 40%, 60%, 80%, and 100% barley.

Regarding the total soluble solids content of rice snacks, RSB0 exhibited the lowest value at 0.30 °Brix, and this value increased significantly with greater barley addition, reaching a maximum of 1.20 °Brix in RSB100. The primary components contributing to total soluble solids in rice snack (RSB0) are presumed to be amylose, a water‐soluble substance derived from cooked rice, as well as dextrin, maltose, and glucose produced by the degradation of amylose during high‐temperature heating (Kang and Lho 1998). The β‐glucan content of barley, a water‐soluble fiber composed by glucose, is reported to vary markedly, ranging from less than 2% to over 10% (Geng et al. 2022), and is higher comparing with rice, wheat, and rye (Lante et al. 2023). These observations support the hypothesis that the increasing total soluble solids content in rice snack with higher barley content results from the higher levels of β‐glucan in barley compared to rice.

The pH of rice snacks ranged from 5.12 to 6.58 and decreased significantly as the proportion of barley increased. RSB0 exhibited the highest pH at 6.58, while RSB100 had the lowest pH at 5.12 (Table 1). Hwang and Moon (2021) reported that the pH values for rice and barley snacks were 6.63 and 5.38, respectively, indicating that rice snack has a higher pH than barley snack, which aligns with the findings of this study. The crude ash content of rice snacks was lowest at 0.24% for both RSB0 and RSB20, and highest at 0.61% for RSB100 (Table 1). Hwang and Moon (2021) reported that the crude ash content of rice and barley snacks was 0.42% and 0.82%, respectively, which is higher than both RSB0 and RSB100, although the trend was similar. Given that barley has a higher crude ash content than rice, it appears that the crude ash content increases with the amount of barley added to rice snacks.

The texture of rice snacks is considered one of the most important quality attributes when evaluated as a cracker (Giannoutsos et al. 2023; Yilmaz and Karaman 2017). The hardness of rice snacks is presented in Table 1. Hardness increased as the proportion of barley was raised, with RSB100 exhibiting the highest value. Previous studies have reported that the hardness of wheat crackers increases when different types of fiber are added (Yilmaz and Karaman 2017).

In the colorimetric analysis, RSB0 showed the highest L* values, indicating it was the brightest (Table 1). As barley content increased, both L* and b* values declined significantly. Meanwhile, the a* values of rice snacks tended to increase as the amount of barley increased (*p* < 0.05). The color difference (△E*) represents the combined change in L*, a*, and b* values, quantifying deviation from the standard sample's color. As a greater amount of barley was incorporated, △E* increased markedly, reflecting a visible color difference. The decrease in lightness (*L* values*) with increased barley content is likely due to the acceleration of the Maillard reaction promoted by the higher levels of reducing sugars and amino compounds including proteins in barely (Geng et al. 2022). This reaction is further enhanced during high‐temperature processing (205°C), leading to increased formation of melanoidins (Hwang and Moon 2021). In addition, total polyphenol content and polyphenol oxidase (PPO) activity have been reported as key factors influencing the lightness (L* value) of barley‐based products (Quinde et al. 2004). Ou et al. (2019) demonstrated that polyphenols play a critical role in determining the color profiles of various baked goods, including cookies, biscuits, muffins, and cakes. Free phenolic acids, such as caffeic acid, have been shown to intensify browning during high‐temperature processing (Kohyama et al. 2009). Specifically, the mineral components within the ash can directly darken the product's color and also act as catalysts that accelerate both enzymatic and non‐enzymatic browning reactions during processing (Quinde et al. 2004). These factors collectively contribute to the darker appearance of snack samples with higher barley concentrations.

### 3.2 Phenolic Compounds

The phenolic profiles of the rice snacks, including total phenolics, total flavonoids, and five phenolic acids, are presented in Table 2. Total phenolic contents increased significantly with increasing barley percentages (*p* < 0.05), ranging from 3.99 to 12.07 mg GAE/g for RSB0 and RSB100, respectively. Total flavonoid contents showed a similar trend, increasing 4.25‐fold from 0.68 mg RE/g (RSB0) to 2.89 mg RE/g (RSB100). These results indicate that barley incorporation is a major contributor to the phenolic content of the formulated snacks. These level‐dependent increases align with Sharma and Gujral (2014), who observed a 3.28‐ to 3.86‐fold increase in total phenolics and flavonoid contents, respectively, as barley was substituted from 0% to 100% in cookies. Similarly, Kumari et al. (2018) reported an increase in total phenolic content from 1.13 to 1.36 mg GAE/g when rice flour was substituted with 0%–40% barley flour.

**TABLE 2:** Phenolic compound contents of rice snacks fortified with barley.

	RSB0	RSB20	RSB40	RSB60	RSB80	RSB100
Total phenolics (mg GAE/g)	3.99 ± 0.07^f^	4.97 ± 0.01^e^	5.43 ± 0.05^d^	7.63 ± 0.01^c^	9.77 ± 0.02^b^	12.07 ± 0.09^a^
Total flavonoid (mg RE/g)	0.68 ± 0.01^f^	1.19 ± 0.01^e^	1.36 ± 0.01^d^	1.57 ± 0.01^c^	2.07 ± 0.01^b^	2.89 ± 0.01^a^
*p*‐Hydroxyl benzoic acid (µg/g)	2.50 ± 0.58^d^	5.19 ± 1.21^d^	13.10 ± 3.18^c^	20.25 ± 4.78^b^	23.07 ± 3.06^ab^	25.97 ± 3.48^a^
Syringic acid (µg/g)	3.81 ± 0.24^e^	4.92 ± 0.13^e^	6.41 ± 0.94^d^	9.76 ± 0.35^c^	16.72 ± 1.09^b^	21.86 ± 1.10^a^
Caffeic acid (µg/g)	7.49 ± 0.82^e^	9.18 ± 0.47^e^	11.55 ± 0.35^d^	14.32 ± 1.39^c^	22.96 ± 1.68^b^	30.95 ± 1.67^a^
*p*‐Coumaric acid (µg/g)	3.36 ± 0.51^e^	5.51 ± 1.06^de^	6.89 ± 0.64^d^	12.46 ± 1.51^c^	17.56 ± 2.54^b^	21.99 ± 1.61^a^
Ferulic acid (µg/g)	41.77 ± 0.28^f^	56.63 ± 6.69^e^	67.94 ± 4.30^d^	76.66 ± 3.36^c^	98.98 ± 5.71^b^	116.99 ± 3.75^a^
Total phenolic acid (µg/g)	58.92 ± 2.08^f^	81.44 ± 7.37^e^	105.89 ± 2.26^d^	133.43 ± 8.63^c^	179.28 ± 5.68^b^	217.75 ± 9.34^a^

*Note*: Results are expressed as average standard deviation (*n* = 3). Different letters in the same row indicate significant differences (*p* < 0.05).

RSB0, RSB20, RSB40, RSB60, RSB80, and RSB100: Rice snacks fortified with 0%, 20%, 40%, 60%, 80%, and 100% barley.

Notably, the total phenolic content of RSB0 was higher than values reported for rice crackers 0.17 and 0.61 mg GAE/g (Rico et al. 2019; Mir et al. 2017). In addition, barley snacks processed using twin‐screw extrusion have been reported to exhibit total phenolic contents ranging from 1.90–3.28 mg GAE/g at 150°C and 1.79–2.88 mg GAE/g at 180°C (Punia Bangar et al. 2022), which appear lower than those observed in the present study. Such differences may be partly attributed to variations in moisture content and processing conditions. Although the moisture content of the previously reported extrusion snacks was not reported, previous study on cereal extrusion have indicated typical moisture levels of approximately 3%–6% (Theng et al. 2025), which are substantially higher than those of the present samples (0.40%). This suggests that moisture differences may have contributed to the observed phenolic values in the snacks. In the present study, high‐temperature baking at 205°C for 4 min may have promoted the release of bound phenolic acids, while the subsequent reduction in moisture content likely resulted in a concentration effect, contributing to higher measured phenolic values.

The concentrations of five phenolic acids increased significantly as the barley percentage rose (Table 2). Specifically, *p*‐hydroxybenzoic, syringic, and caffeic acids in the rice snacks ranged from 2.50–25.97, 3.81–21.86, and 7.49–30.95 µg/g, with increasing barley percentages. While these acids are present in trace amounts in raw rice (Goufo and Trindade 2014), our results confirm that increasing barley percentages consistently enhances their levels in the final product. Consistent with previous reports that caffeic acid exceeds *p*‐hydroxybenzoic and syringic acids in rice (Goufo and Trindade 2014; Ma et al. 2022), our snacks also exhibited higher caffeic acid content.

*p*‐Coumaric and ferulic acids, recognized as predominant phenolic acids in cereal grains (Ma et al. 2022; Geng et al. 2022; Raj et al. 2023), also rose significantly with barley addition. The *p*‐coumaric acid contents in rice snacks were found to range from 3.36 to 21.99 µg/g (Table 2). Ferulic acid was the most prevalent constituent in rice snacks, ranging from 41.77 to 116.99 µg/g (Table 2). Ferulic acid is the predominant phenolic acid in the endosperm, bran, and whole‐grain rice, constituting 56%–77% of the total phenolics (Goufo and Trindade 2014). Previous studies have indicated that barley generally contains higher levels of both free and bound *p*‐coumaric and ferulic acids than rice (Harukaze et al. 1999; Abdel‐Aal et al. 2012; Dvořáková et al. 2008). Consequently, increasing the barley substitution levels led to a marked increase in phenolic acids, as evidenced by a 2.8‐fold rise in ferulic acid content in RSB100 compared to RSB0, with *p*‐coumaric acid exhibiting a similar increasing trend (Table 2).

Overall, the total content of these five free phenolic acids increased from 58.92 µg/g (RSB0) to 217.75 µg/g (RSB100). These elevated levels can be explained by the inherently higher phenolic profile of barley, as well as the possible breakdown of cell‐wall‐bound phenolic acids during the high‐temperature heating process. This study demonstrates that barley‐fortified snacks, with their enriched free phenolic acid profiles, possess superior potential as functional snacks compared to reported rice‐based products (Blandino et al. 2022; Lang et al. 2020). However, the present HPLC analysis was limited to changes in the quantified phenolic acid profile and did not allow structural identification of potentially new products formed during snack processing.

Figure 2 displays the UV absorption spectra of standard phenolic acids. The phenolic acids include *p*‐hydroxybenzoic and syringic acids from the hydroxybenzoic acid derivatives, and *p*‐coumaric, caffeic, and ferulic acids from the hydroxycinnamic acid derivatives. UV absorption peaks around 235 nm were observed for both the standard phenolic acids and the phenolic compounds extracted from rice snacks. Hydroxybenzoic acid derivatives generally have a C6–C1 structure derived directly benzoic acid (Goufo and Trindade 2014). They exhibit a *λ*max within the 200–280 nm range, with strong absorption at 280 nm (Mutiarahma et al. 2021). The *λ*max for the standards of *p*‐hydroxybenzoic and syringic acids are 255 nm (Figure 2A) and 275 nm (Figure 2B), respectively.

**FIGURE 3:**
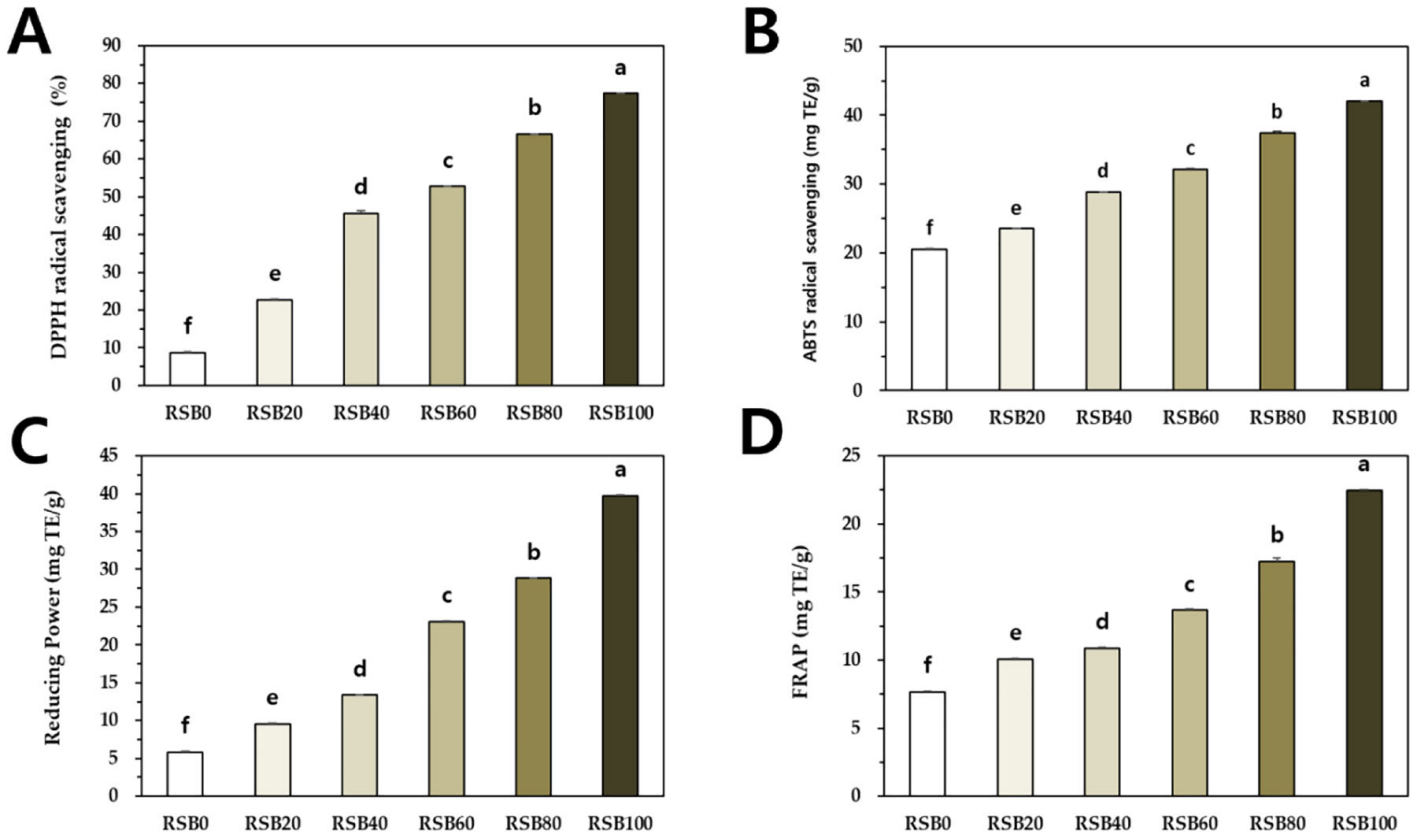
DPPH radical scavenging activity (A), ABTS radical scavenging activity (B), reducing power (C), FRAP (D) of rice snacks fortified with barley. RSB0, RSB20, RSB40, RSB60, RSB80, and RSB100: Rice snacks fortified with **0%, 20%, 40%, 60%, 80%, and 100%** barley. Different letters above the bars indicate significant differences (*p* < 0.05).

Hydroxycinnamic acid derivatives feature a carbon–carbon double bond (─CH═CH─) between the benzene ring and the COOH group. The *λ*max for caffeic, *p*‐coumaric, and ferulic acids are 323, 309, and 322 nm, respectively, generally clustering aound 320 nm (Figure 2C‐E). The absorption around 320 nm results from the electron traansitions from the highest occupied molecular orbital (HOMO) to the lowest unoccupied molecular orbital (LUMO). This phenomenon indicates a *π*–π* transition, with electron density delocalized on the benzene ring and the adjacent carbon chain (Belay and Gholap 2009).

The UV absorption spectra of phenolic acids in the rice snack samples (RSB20 and RSB100) are presented, with RSB100 spectrum at the top and RSB20 spectrum at the bottom (Figure 2A‐E). The increased absorbance observed in RSB100 spectra is attributed to the increase in the concentration resulting from the barley addition. In addition, since RSB100 extract used in the experiment was diluted twofold with methanol, the actual absorbance of each phenolic acid in RSB100 spectrum would be twofold higher than in Figure 2A‐E. In particular, the absorbance of *p*‐coumaric and ferulic acids in RSB20 and RSB100 (Figure 2D,E) was higher than that of *p*‐hydroxybenzoic and syringic acids at their respective maximum wavelengths. The UV absorption is related to chromophores with conjugated double bonds within the molecule (Shukla et al. 2012). The chromophores of phenolic compounds such as *p*‐coumaric and ferulic acids have a high capacity to absorb the UV light (Mutiarahma et al. 2021) at 309 and 322 nm, respectively, thereby increasing absorbance of coumaric and ferulic acids.

### 3.3 Antioxidant Activities

Antioxidant activities of rice snack samples are shown in Figure 3A‐D. DPPH and ABTS free radical scavenging activities (Figure 3A,B), reducing power (Figure 3C), and FRAP (Figure 3D) of rice snacks all increased in a level‐dependent manner as barley percentage rose from 0% to 100% (*p* < 0.05). This trend can be attributed to the higher content of total phenolics, total flavonoids, and phenolic acids in barley when compared to rice (Table 2). Phenolic compounds act as the primary antioxidants in rice and barley. Among the antioxidant activity assays used in this study, the DPPH assay is rapid and simple, but it has limited applicability in aqueous systems, whereas the ABTS assay is applicable to both hydrophilic and lipophilic systems and can be performed rapidly. However, both methods rely on non‐physiological radicals (Munteanu and Apetrei 2021). FRAP and the reducing power assay both assess antioxidant capacity based on the electron‐donating ability of the sample. These two assays differ in the reagents used and in the method of detecting iron ions. In addition, the FRAP assay is additionally limited by its requirement for an acidic reaction environment (Munteanu and Apetrei 2021). Therefore, four antioxidant assays were employed in this study to evaluate antioxidant capacity under in vitro conditions encompassing different solubility characteristics and reaction environments.

**FIGURE 4:**
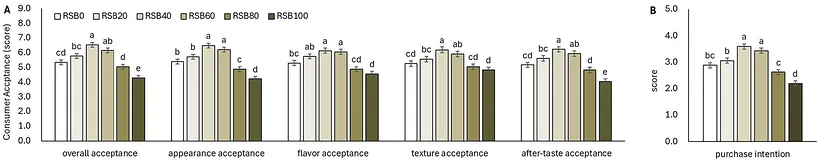
Consumer acceptance (A) and purchase intention (B) of rice snacks fortified with barely. RSB0, RSB20, RSB40, RSB60, RSB80, and RSB100: Rice snacks fortified with **0%, 20%, 40%, 60%, 80%, and 100%** barley. Different letters above the bar indicate significant differences (*p* < 0.05).

Our observations align with the results of Hwang and Moon (2021), who demonstrated that 100% barley snacks (59.53%) possess significantly greater DPPH radical scavenging ability compared to 100% rice snacks (43.71%). DPPH free radical scavenging activity significantly increased from 1.35% to 10.1% and 12.6% as the substitution level of barley powder increased from 0% to 20% and 40%, respectively (Aly et al. 2021). Importantly, the DPPH radical scavenging activity of 100% barley snack in this study (77.34%) appears higher than values reported in previous investigations. While genotypic variations among barley cultivars undoubtedly contribute to these differences (Goufo and Trindade 2014; Dvořáková et al. 2008; Zhu et al. 2015; Abdel‐Aal et al. 2012), the superior antioxidant capacity in our study also likely stems from the specific processing conditions. Particularly, the high‐temperature, short‐time, and low‐moisture processing conditions employed in this study, which may have contributed to the formation of Maillard reaction products (MRPs), thereby influencing antioxidant capacity.

Similarly, Sharma and Gujral (2014) reported that the antioxidant capacity significantly increased as the proportion of barley flour rose in cookies. Notably, cookies formulated with 100% barley displayed a reducing power four times greater than that of control wheat cookies, a result attributed to the inherently higher reducing power of barley flour, as well as the formation of MRPs during baking (Sharma and Gujral 2014). Furthermore, FRAP values for barley‐substituted crackers were significantly higher at a 35% barley content compared to 15% (Gangopadhyay et al. 2019). This suggests that increasing the barley proportion is an effective strategy for enhancing the functional potential of cereal‐based snacks.

### 3.4 Consumer Acceptance

**FIGURE 5:**
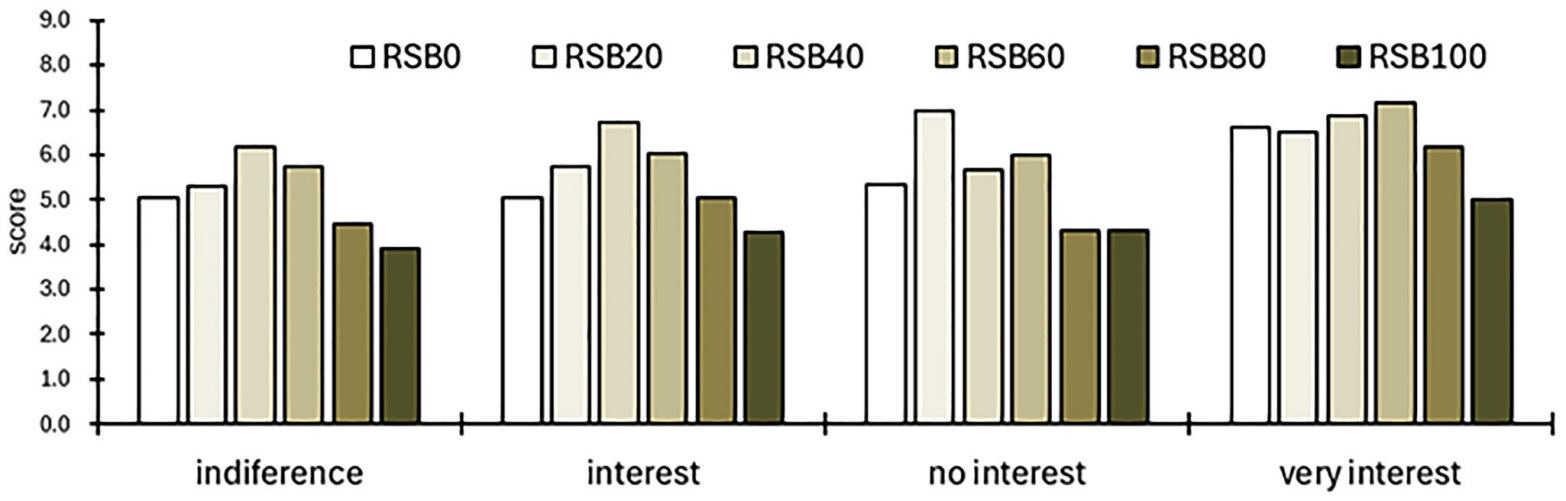
Consumer acceptance results according to the level of consumer interest in crunchy foods. RSB0, RSB20, RSB40, RSB60, RSB80, and RSB100: Rice snacks fortified with 0, 20, 40, 60, 80, and 100% barley. Overall differences among consumer groups were evaluated using MANOVA based on Wilks' lambda with Rao's approximation (*p* < 0.05).

**FIGURE 6:**
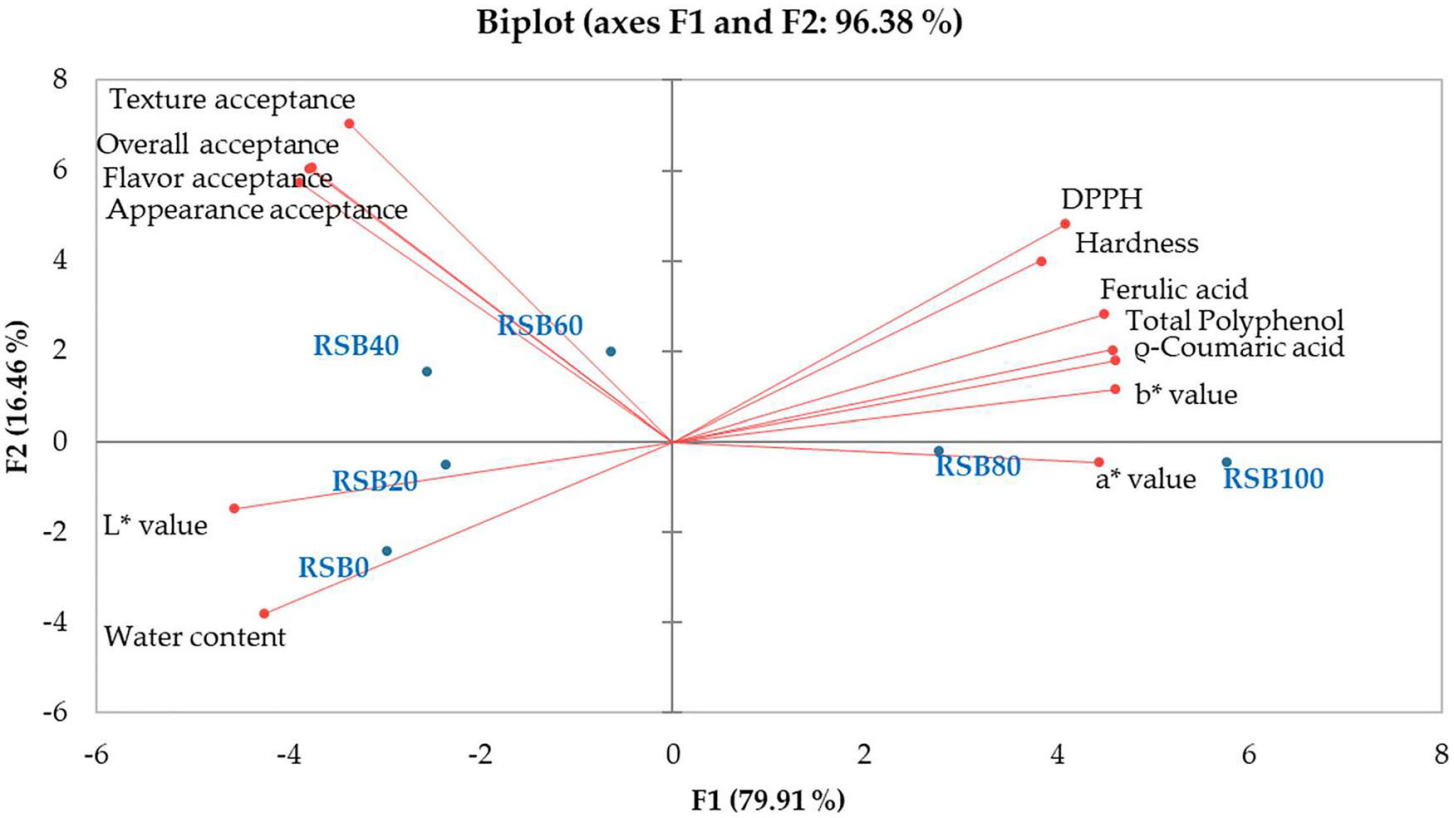
Principal component analysis (PCA) of rice snacks fortified with barely. RSB0, RSB20, RSB40, RSB60, RSB80, and RSB100: Rice snacks fortified with **0%, 20%, 40%, 60%, 80%, and 100%** barley.

The consumer acceptance test was performed to identify the optimal concentration of barley addition in the rice snack samples. The results of consumer acceptance and purchase intention are presented in Figure 4. Overall acceptance was significantly higher for RSB40 (6.49 points) and RSB60 (6.21 points), but declined with further increases in barley addition (Figure 4A). This result indicates that barley addition positively affects consumer overall acceptance up to a 60% ratio. Consumer acceptance according to the level of interest in crunchy snacks is presented in Figure 5. Consumers with high interest showed the highest overall acceptance for RSB60, those with interest or indifference for RSB40, and those with no interest for RSB20. Acceptance for appearance, flavor, texture, and aftertaste in rice snacks exhibited trends similar to overall acceptance. These findings indicate that barley additions of 40%–60% are optimal for achieving desirable consumer acceptance in terms of appearance, flavor, texture, and aftertaste, while additions of 80% or more are less suitable from a consumer acceptance standpoint. Purchase intent for barley‐fortified rice snacks was assessed using a 5‐point Likert scale, with significantly higher scores for RSB40 (3.59 points) and RSB60 (3.44 points), and notably lower scores for RSB80 (2.62 points) and RSB100 (2.20 points) (Figure 4B). Hęś et al. (2014) found that the bitterness in barley‐containing bread is related to polyphenols and flavonoids. To further explore the relationship between barley addition and quality, including physicochemical characteristics, antioxidant properties, and consumer acceptance in the rice snacks, PCA results are shown in Figure 6.

Antioxidant properties related to DPPH, ferulic acid, total polyphenol, and color indices (redness a* value and yellowness b* value) together with hardness, loaded in the positive (+) direction of PC1, exhibiting a positive correlation with RSB80 and RSB100. Conversely, the negative (−) direction of PC1 and the positive (+) direction of PC2 included RSB40 and RSB60, as well as overall acceptance, flavor acceptance, and texture acceptance. The negative (−) direction of both PC1 and PC2 was associated with RSB0 and RSB20, along with lightness (L* value), and moisture content. These findings reveal that as barley addition increases, antioxidant activities and color characteristics (higher redness and yellowness) are more pronounced in rice snacks containing higher barley content, while consumer acceptance is more closely linked to RSB40 and RSB60.

**FIGURE 7:**
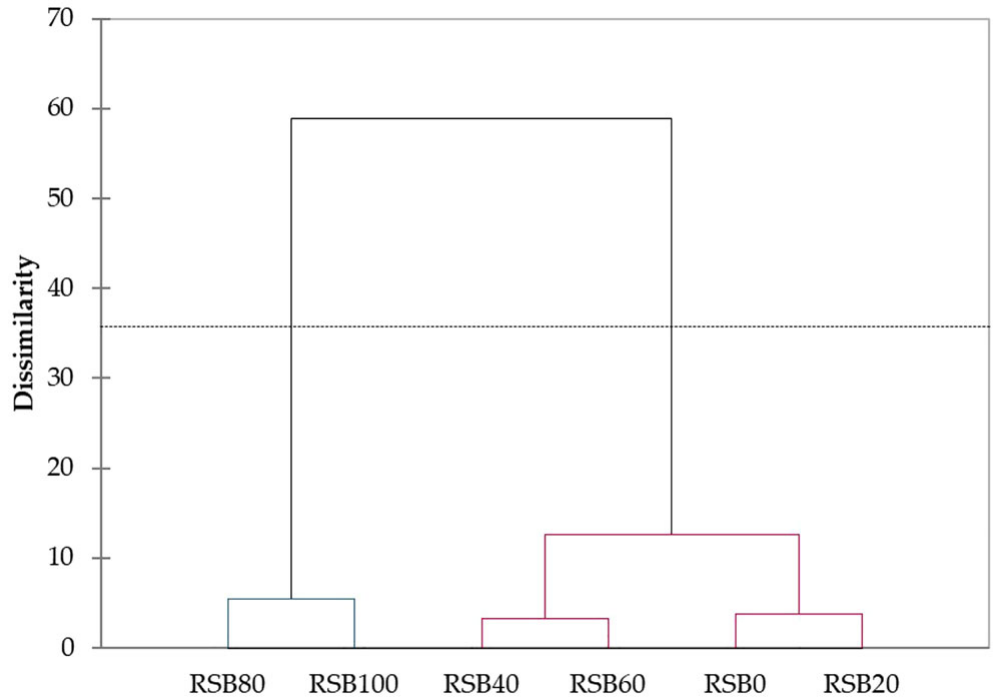
Agglomerative hierarchical clustering (AHC) dendrogram of rice snacks fortified with barely based on consumer acceptance test. RSB0, RSB20, RSB40, RSB60, RSB80, and RSB100: Rice snacks fortified with **0%, 20%, 40%, 60%, 80%, and 100%** barley.

The AHC analysis (Figure 7) confirms the trends observed in the PCA (Figure 6). The analysis categorized the six rice snack samples fortified with barley into three distinct clusters: Cluster 1 consisted of two samples (RSB80 and RSB100) that were associatewith higher levels of antioxidant compounds, enhanced antioxidant properties, and elevated color values for redness and yellowness. Cluster 2, including two samples (RSB40 and RSB60), was characterized primarily by their sensory attributes. Cluster 3 comprised two samples (RSB0 and RSB20). These rice snacks were distinguished by higher lightness (L* value), greater moisture content. The positioning of these groups supports the notion that consumer acceptance may surpass physicochemical or antioxidant properties when there are marked differences in product characteristics.

## 4 Conclusions

This study demonstrated that barley fortification influences the physicochemical properties, phenolic composition, antioxidant activities, and consumer acceptance of rice snacks. Rice snacks containing 40%–60% barley exhibited higher consumer acceptance while maintaining enhanced phenolic content and antioxidant activities compared to those without barley. The observed increases in antioxidant activities were associated with higher levels of phenolic compounds, particularly ferulic acid. However, further increases in barley percentage adversely affected textural and sensory attributes, including hardness and color.

## Author Contributions

**Il Sook Choi**: conceptualization, methodology, formal analysis, writing – original draft, supervision, funding acquisition. **Eun Jung Kwak**: investigation, resources, data curation, writing – original draft, writing – review and editing, visualization.

## Funding

This paper was supported by Wonkwang University in 2024.

## Conflicts of Interest

The authors declare no conflicts of interest.
